# Can increasing the number of excitations (NEX) in late gadolinium enhancement (LGE) imaging prove beneficial in patients who can not hold their breath for the duration of the scan time?

**DOI:** 10.1186/1532-429X-14-S1-T10

**Published:** 2012-02-01

**Authors:** June Yamrozik, Mark Doyle, Ronald B Williams, Geetha Rayarao, Diane A Vido, Moneal Shah, Robert W Biederman

**Affiliations:** 1 Allegheny General Hospital, Pittsburgh, USA

## Summary

Image quality greatly improves using a LGE sequence with 4 NEX in patients that cannot hold their breath.

## Background

It is imperative that patients hold their breath for Cardiac MRI imaging.

Parallel imaging can be implemented on steady state free precession (SSFP) imaging to reduce scan time. However, for LGE imaging parallel imaging is not available. Manipulation of other parameters such as matrix, views per segment can be utilized but scan times are still too long for patients that cannot hold their breath. Increasing the NEX will increase scan time but will decrease respiratory motion. Perhaps, a free breathing technique can be mastered instead of trying various ways for a patient to hold their breath.

### Hypothesis

We propose that by doubling the NEX on LGE imaging, free breathing can be utilized and that image quality will improve.

## Methods

A total of 12 patients were imaged on a GE CV/i Excite Version 12, 1.5 T system (GE, Milwaukee, WI). The LGE sequence utilized was a 2D Gradient Echo IRP (FGR with inversion recovery prep). The standard NEX parameter which is 2 was utilized on all 12 patients for breath hold imaging. The NEX parameter was increased to 4 which doubled scan time and the 12 patients were re-imaged without holding their breath. THree observers rated the image quality, from a scale of 1 to 4. Four(4) being the highest image quality and one(1) being the lowest.

## Results

**Figure 1:**
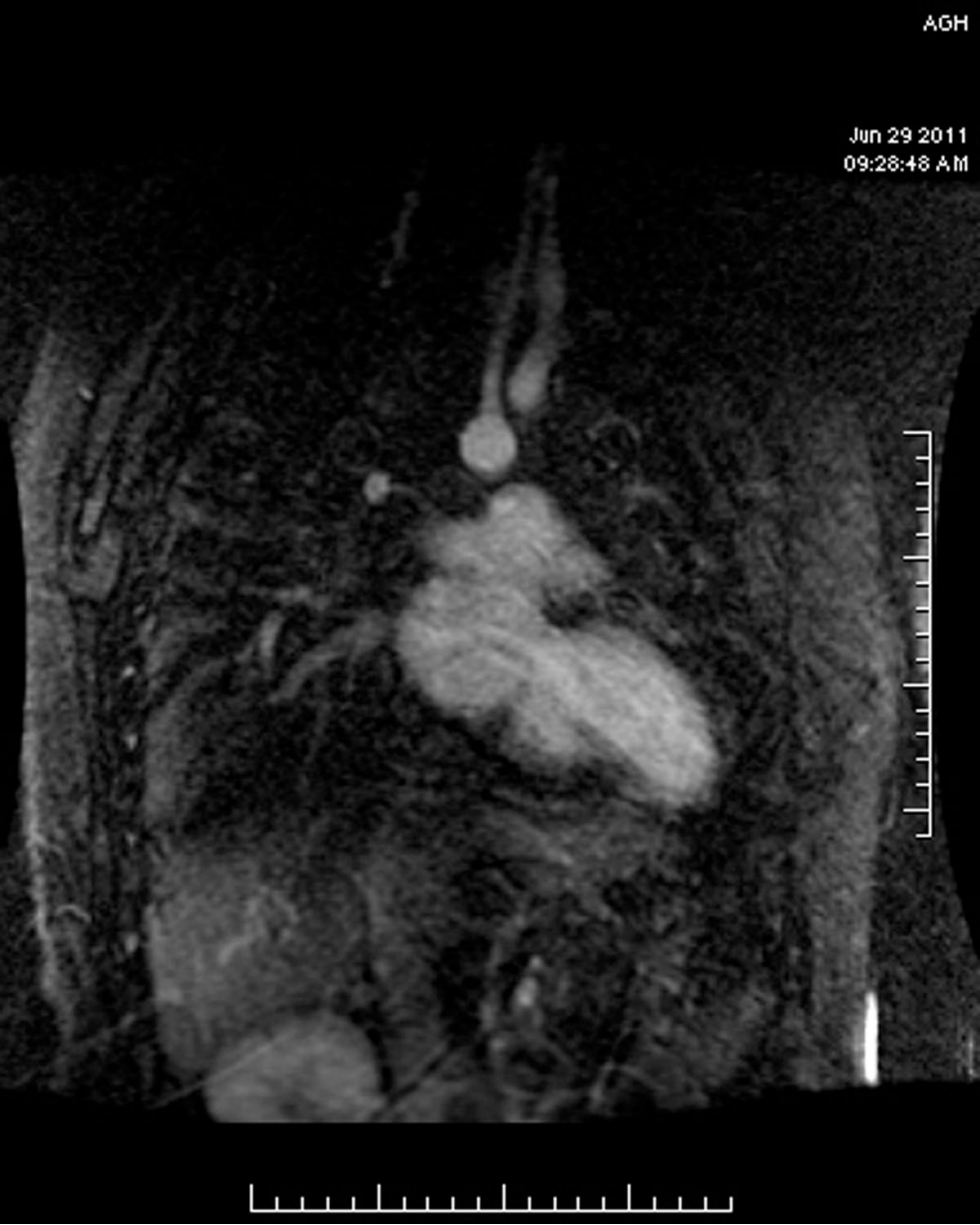
The patient was unable to hold their breath using the standard FGR sequence with 2 NEX.

**Figure 2:**
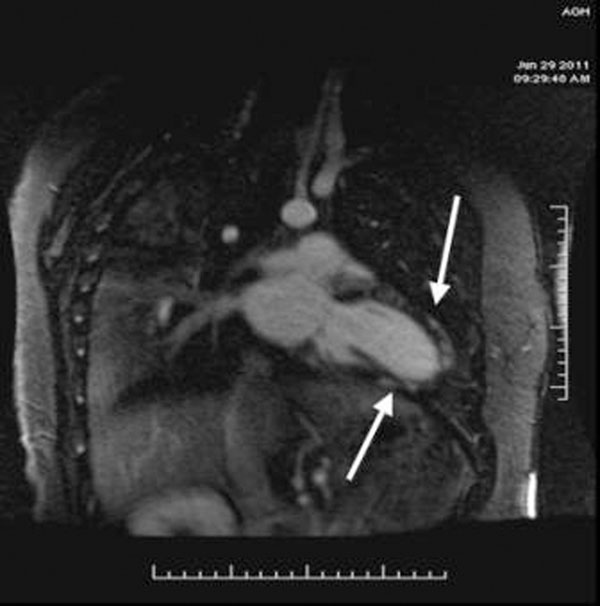
Using 4 NEX and free breathing image quality is greatly improved and an important diagnosis of myocarditis is made

Of the 12 patients imaged, 3 were unable to hold their breath, 2 had difficulty holding their breath and 7 had no difficulty suspending their breath. The CNR ratio remained essentially unchanged between 2 and 4 NEX (4.1 ± 1.4, 4.4± 1.6). Interobserver results showed that the best image quality was obtained on those who could hold their breath (3.4±.5) while those who could not hold their breath had the lowest quality (2.1±.8). However, performing 4 NEX allowed the quality of those who could not hold their breath to increase (3.6±.5, p<0.01), while those comfortable holding their breath trended to decrease in quality when 4 NEX were used (3.0±.9, p=0.06). Those with partial ability to hold breath had the best results with 4 NEX (2.8±.7 vs. 3.6±.5, p=0.1). (Fig 1 &2).

## Conclusions

Interestingly, increasing the NEX to 4 is advantageous when imaging a patient that cannot hold their breath. This information is useful, especially for those systems that do not have a LGE sequence that can accommodate patients that cannot hold their breath.

## Funding

None.

